# Physiological Profile, Mediterranean Diet Adherence and Supplement Use in Recreational Adults Engaged in Contemporary Gym-Based Exercise Modalities

**DOI:** 10.3390/sports14090368

**Published:** 2026-08-25

**Authors:** Dimitrios Dragoumis, Fotios Mavrovouniotis, Evangelia Kouidi, Nikolaos Koutlianos

**Affiliations:** Sports Medicine Laboratory, Aristotle University of Thessaloniki, 541 24 Thessaloniki, Greecekouidi@phed.auth.gr (E.K.); koutlian@phed.auth.gr (N.K.)

**Keywords:** CrossFit, Pilates, combined training, VO_2_max, ventilatory anaerobic threshold, body composition, handgrip strength, flexibility, Mediterranean diet, dietary supplements

## Abstract

Physical exercise is a major determinant of health and functional capacity, yet direct comparisons among contemporary gym-based exercise modalities remain limited. This cross-sectional study compared the fitness, dietary, and supplement-use profile of 148 healthy recreational adults (72 men and 76 women; aged 18–45 years) engaged in mixed aerobic-resistance training (*n* = 52), Pilates (*n* = 46), or CrossFit (*n* = 50). Participants underwent cardiopulmonary exercise testing to determine the maximal oxygen uptake (VO_2_max) and ventilatory anaerobic threshold (VAT). Body composition, handgrip strength, flexibility, Mediterranean diet adherence, and supplement use were also assessed. Participants in CrossFit and mixed training showed higher VO_2_max and lower fat mass than the Pilates participants. Pilates showed higher VAT expressed as a percentage of VO_2_max; however, this finding should be interpreted as a relative submaximal profile rather than evidence of superior overall aerobic fitness. Mixed training and CrossFit also showed more favorable body composition profiles, while mixed training demonstrated superior handgrip strength. Flexibility did not differ significantly among exercise modalities. Mediterranean diet adherence showed limited and sex-specific differences, which were attenuated after adjustment for age and training age. Overall, exercise modality was associated with distinct physiological and nutritional characteristics, with CrossFit and mixed training showing greater cardiorespiratory fitness and more favorable body composition characteristics, whereas Pilates was linked to a different submaximal exercise profile and specific lifestyle-related patterns. These findings should be interpreted with caution given the cross-sectional design and the possibility of self-selection bias.

## 1. Introduction

Modern lifestyle is characterized by increased sedentary behavior and reduced physical activity. This has led to growing interest in exercising in organized gym environments, where different training modalities with varying intensity and focus are offered. In this context, the assessment of physical fitness through reliable and functionally meaningful indicators is considered essential for both the scientific documentation of exercise-induced adaptations and the rational prescription of physical activity programs [1]. This approach is fully aligned with contemporary exercise testing and prescription frameworks proposed by the American College of Sports Medicine and with broader international physical activity recommendations [2,3].

Among the fitness indicators, maximal oxygen uptake (VO_2_max) is recognized as one of the most powerful and independent predictors of cardiorespiratory health and overall functional capacity. Higher VO_2_max values reflect adaptations such as increased cardiac output, improved oxidative capacity of skeletal muscles, and more efficient oxygen utilization. These mechanisms are directly associated with a reduced risk of cardiovascular disease and mortality. Recent evidence also continues to support the metabolic and clinical significance of cardiorespiratory fitness and VO_2_max responsiveness across exercise interventions [4,5].

Beyond VO_2_max, ventilatory anaerobic threshold (VAT), as well as indices of muscular strength, flexibility, and body composition, provide a multidimensional and more comprehensive picture of physical fitness, allowing for a broader evaluation of functional performance and adaptation to exercise. In applied exercise testing, these variables are particularly meaningful when interpreted within standardized assessment frameworks such as treadmill exercise testing protocols and formal exercise prescription guidelines [2,6].

In recent years, exercise in organized gym environments has diversified substantially, with the emergence and wide dissemination of different training modalities, such as CrossFit, Pilates, and mixed aerobic-resistance training, which are characterized by different training stimuli, intensity levels, and training goals [7,8,9,10]. CrossFit, as a form of high-intensity functional training, has expanded rapidly worldwide. Its training philosophy incorporates core principles of exercise science, such as progressive overload, variability, multi-joint movement patterns, and high-intensity training within a structured time frame, with the aim of improving multiple physical domains [7,11]. Participating in CrossFit training has been associated with improvements in cardiorespiratory fitness, body composition, and overall physical performance, although the literature also emphasizes the need for careful supervision and appropriate programming [12,13,14]. At the same time, Pilates is a widely practiced exercise method of lower to moderate intensity, with emphasis on movement control, trunk stabilization, and flexibility. Research has shown that Pilates may contribute to improvements in physical fitness, flexibility, functional stability, and quality of life, particularly in healthy and rehabilitation populations [8,15,16,17]. In addition, mixed training programs, which integrate aerobic exercise and resistance training, are widely recommended by international organizations as an effective strategy for improving cardiorespiratory fitness, muscular strength, and body composition [1]. Concurrent training of aerobic and resistance exercise patterns has been repeatedly discussed as a balanced and effective model for producing broad physiological adaptations, although the interaction between endurance and strength stimuli may depend on exercise sequencing, training load, and participant characteristics [9,18]. Clinical and applied studies also support the effectiveness of aerobic-resistance combinations for functional health and cardiometabolic improvement.

Despite the widespread popularity of the aforementioned workout types, comparative studies simultaneously examining the cardiorespiratory, neuromuscular, and functional adaptations induced by these exercise modalities remain limited, especially when multiple fitness indicators and sex-related differences are taken into account [7,19]. More specifically, to the best of our knowledge, limited research appears to exist on a direct head-to-head comparison of CrossFit, Pilates, and mixed aerobic-resistance training within the same study design using a multidimensional set of outcomes including VO_2_max, VAT, body composition, muscular strength, and flexibility in recreational adults. In parallel, behavioral and motivational aspects may also influence long-term participation and adaptation, especially in modalities such as CrossFit and organized gym-based exercise [7].

Therefore, the aim of the present study was to compare the fitness, dietary, and supplement-use profile of recreational adults engaged in CrossFit, Pilates, and mixed aerobic-resistance training while accounting for sex-related differences. In this respect, the study seeks to address an evident gap in the current literature by providing a direct comparative cross-sectional analysis of these three widely practiced gym-based exercise modalities within an integrated health and fitness framework.

## 2. Materials and Methods

### 2.1. Study Design

This study followed a cross-sectional comparative design to evaluate differences in physiological, functional, and lifestyle characteristics among adults participating in three contemporary gym-based exercise modalities: CrossFit, Pilates, and mixed aerobic-resistance training. The primary outcomes were physiological and functional characteristics, including cardiorespiratory fitness, body composition, muscular strength, and flexibility. Secondary outcomes included Mediterranean adherence and dietary supplement use.

### 2.2. Participants

Participants were recruited from ten fitness facilities located in Thessaloniki and Chalkidiki, Greece. Facilities were selected through random electronic allocation according to exercise modality. Five gyms offered all three exercise modalities, three specialized in Pilates, and two operated as CrossFit facilities. Individuals were informed about the study procedures in their exercise setting and volunteered after screening for eligibility.

Inclusion criteria were age between 18 and 45 years, healthy status, and regular participation in the same exercise modality at least three times per week for more than twelve months [20,21,22,23]. Participants were eligible only if they were systematically and exclusively engaged in the exercise modality examined in the group to which they were assigned [24]. This information was verified during the screening process based on participant self-report. Exclusion criteria included known cardiopulmonary, metabolic, or musculoskeletal disease, use of medication affecting physical performance, injury within the last six months, as well as regular participation in another structured exercise modality and former competitive athletic participation.

An a priori power analysis was conducted using G*Power (version 3.1.9.7) based on the F-test family and the fixed-effects ANCOVA option for main effects and interactions. Assuming an effect size of (f = 0.30), an alpha level of 0.05, a statistical power of 0.90, six design cells arising from the 3 (exercise modality: mixed training, Pilates, and CrossFit) × 2 (sex: men and women) factorial design, two covariates, and two numerator degrees of freedom, the minimum required total sample size was 144 participants (actual power = 0.9005). The final sample comprised 148 healthy adults (72 men and 76 women), exceeding the required sample size.

### 2.3. Ethical Approval

The study was approved by the Research Ethics and Deontology Committee of Aristotle University of Thessaloniki (protocol no. 163583/2023) and was conducted according to the principles of the Declaration of Helsinki. All participants were fully informed about the purpose and procedures of the study and provided written informed consent prior to participation.

### 2.4. Training Session Structure by Exercise Modality

Across all three exercise modalities, sessions were supervised by certified and qualified coaches, performed at least three times per week, and generally organized into warm-up, main exercise, and cool-down phases. Modality-specific duration, content, and intensity characteristics are described below.

The mixed training sessions combined aerobic exercise and resistance training and each session lasted approximately 60–70 min. Initially, a brief warm-up was performed, including light aerobic activity and dynamic mobilization exercises, followed by a moderate-intensity aerobic segment on a treadmill, elliptical trainer, or stationary bicycle. Subsequently, a full-body resistance training component was implemented using free weights, bodyweight exercises, and auxiliary equipment such as suspension straps (TRX) and kettlebells. The resistance component was based primarily on multi-joint exercises targeting the lower limbs, trunk, and upper body, often organized in a circuit-based format, whereas the session concluded with static stretching and gentle breathing exercises to facilitate gradual recovery.

The CrossFit training sessions followed a high-intensity interval training (HIIT)-oriented model, with a total duration of approximately 50–70 min. The warm-up aimed to progressively increase body temperature, activate the neuromuscular system, and prepare participants for the specific demands of the workout of the day (WOD). The main exercise phase followed a sequential design, beginning with a structured strength-training component and continuing with a metabolic conditioning segment. The strength component consisted of multi-joint lower- and upper-body exercises incorporating elements of weightlifting, gymnastics, and conditioning drills, with the aim of enhancing muscular strength, stability, coordination, and movement control. This was followed by a high-intensity metabolic phase, commonly implemented in AMRAP (As Many Rounds As Possible) format, in which repeated rounds of demanding exercises were performed under time pressure, combining muscular endurance, aerobic capacity, and anaerobic performance. The session concluded with a brief cool-down period consisting of static stretching and controlled breathing exercises to facilitate gradual physiological recovery.

The Pilates training sessions, lasting approximately 50–60 min each, were structured around the core principles of trunk stabilization, movement control, and breathing-coordinated neuromuscular activation. The warm-up included gentle mobilization, breathing control, and core activation exercises to prepare the neuromuscular system and establish postural alignment and pelvic control. The main part consisted of a progressive sequence of floor-based, quadruped, and seated exercises, often supported by Pilates equipment such as small balls, resistance bands, and rings, with emphasis on the lower limbs, trunk, and upper body. Exercise selection progressed from simpler to more coordinated movement patterns, targeting stability, controlled mobility, muscular endurance, lumbopelvic stabilization, scapular control, and activation of the deep core musculature. Training load and movement complexity were continuously adjusted according to the participant’s muscular and neuromuscular level through variations in exercise type, range of motion, body position, and external props. Training intensity was generally low to moderate and was regulated according to technical execution, postural control, and perceived exertion. The session concluded with gentle static stretching and breathing exercises to promote muscular relaxation and gradual recovery.

Overall, the three exercise modalities were characterized according to their typical session structure and supervised implementation in the participating gyms. However, an identical standardized protocol was not imposed across all participating facilities. Exercise performed in the fitness centers was commonly prescribed at moderate-to-vigorous intensity, with concurrent aerobic–resistance training typically involving aerobic exercise at 40–89% HRR (64–95% HRmax) combined with resistance exercise at approximately 60–70% 1RM. Pilates generally elicited moderate cardiovascular intensity (50–70% HRR; 64–76% HRmax), with CrossFit typically producing vigorous-to-high cardiovascular responses (>90% HRmax) [1,25,26].

### 2.5. Anthropometry and Body Composition

Body composition was estimated by bioelectrical impedance analysis using the BodyStat Quadscan 4000 Touch (Bodystat Ltd., Warwickshire, UK), under standardized resting conditions and according to the manufacturer’s instructions. All assessments were performed within a standardized morning time window (10:00–12:00 h). Furthermore, all measurements were performed under comparable conditions. Participants were instructed to refrain from exercise for 48 h prior to testing, avoid alcohol consumption for at least 24 h, avoid caffeine-containing beverages or substances for at least 4 h before assessment, and avoid food or fluid intake during the preceding 8 h. To minimize potential variation related to menstrual-cycle phase, women who were menstruating on the scheduled assessment day were not evaluated on that day and were reassessed after the completion of menstruation. These procedures were applied to minimize acute pre-test influences on the body-composition-related measurements, in line with commonly used pre-assessment standardization procedures reported in the literature [27,28,29,30,31,32]. Bioelectrical impedance assessment was selected as a practical field-based method for estimating body composition in recreationally active adults and for comparing fat mass, lean mass, and impedance-related variables across training modalities [33]. Variables of interest included fat mass, lean mass, phase angle, and impedance.

### 2.6. Cardiorespiratory Assessment

Cardiorespiratory fitness was assessed using a Trackmaster TMX425CP motorized treadmill (Full Vision Inc., Newton, KS, USA) in conjunction with the COSMED Fitmate Pro metabolic system (COSMED Srl, Rome, Italy). Before each testing session, the Fitmate Pro was operated according to the manufacturer’s instructions, including completion of the built-in O_2_ analyzer check.

Participants performed the standard Bruce treadmill protocol, which consisted of successive 3-min stages with predetermined increases in treadmill speed and grade. Stage 1 was performed at 1.7 mph (2.7 km/h) and a 10% grade, stage 2 at 2.5 mph (4.0 km/h) and a 12% grade, stage 3 at 3.4 mph (5.5 km/h) and a 14% grade, stage 4 at 4.2 mph (6.8 km/h) and a 16% grade, stage 5 at 5.0 mph (8.0 km/h) and an 18% grade, stage 6 at 5.5 mph (8.9 km/h) and a 20% grade, and stage 7 at 6.0 mph (9.7 km/h) and a 22% grade. The test continued until volitional exhaustion, and all 148 participants completed the maximal incremental test to this endpoint [6]. Maximal effort was interpreted according to the accepted physiological criteria including a plateau in oxygen uptake despite increasing workload and the attainment (>90%) of age-predicted maximal heart rate [34]. No separate familiarization or pilot session was performed before the maximal treadmill test; nevertheless, all participants had prior experience with treadmill running. Before testing, participants received standardized verbal instructions regarding the treadmill procedure and the expected progression of the Bruce protocol.

VO_2_max, VAT, heart rate, minute ventilation, and other ventilation-related responses were obtained from the Fitmate PRO v. 2.3 build 13 software output during testing. VAT was determined using the ventilatory-equivalent method, based on the point at which VE/VO_2_ began to increase systematically during incremental exercise. VAT assessment was performed under blinded conditions, as the assessor analyzing the cardiorespiratory test results was unaware of each participant’s exercise-modality group [2].

All cardiorespiratory assessments were performed under standardized pre-test conditions within the same morning time window (10:00–12:00 h), after 48 h without exercise, and at least 4 h without caffeine-containing beverages or substances [28,29].

### 2.7. Muscular Strength and Flexibility Assessment

Muscular strength was assessed using handgrip dynamometry with the Jamar 5030J1 hand dynamometer (Jamar Technologies, Horsham, PA, USA). All assessments were performed by the same evaluator. Before testing, participants received standardized verbal instructions and a practical demonstration of the procedures, first collectively and then individually before each assessment. Three repeated attempts were performed in the strength and flexibility tests, and the best value was retained for analysis. Measurements were obtained for both the left and right hand, and dominant hand grip strength. Handgrip testing was selected as an established and reproducible indicator of upper-body isometric strength and general muscular function [35]. Flexibility was evaluated using the sit-and-reach test [36] with a specially constructed box equipped with a sliding ruler (model 01285; Lafayette Instrument Company, Lafayette, IN, USA), while shoulder flexibility was assessed using the standardized back-scratch test following the established field-based procedures [37]. The sit-and-reach test was selected due to its established criterion-related validity for estimating hamstring and lumbar extensibility [36].

### 2.8. Dietary Profile and Dietary Supplement Assessment

Dietary habits and dietary supplement use were assessed using an anonymous questionnaire administered to the participants. The supplement-related section was developed specifically for the present study as a descriptive data-collection tool and was not based on a previously validated questionnaire. It recorded self-reported dietary supplement and ergogenic aid use, the type of product used, the source of recommendation, the primary reason for use, and smoking status. Accordingly, the supplement-related findings were intended to provide a descriptive overview of the participants’ practices rather than a validated assessment of supplement-use behavior. Adherence to the Mediterranean dietary pattern was assessed separately using the validated MedDietScore [38].

Questionnaire completion was carried out in person at the participants’ exercise setting and required approximately 10 min. The supplement-related items of the questionnaire included questions about commonly used products such as proteins, creatine, vitamins, caffeine, amino acids, and carbohydrates, in line with the broader literature on dietary supplement practices and ergogenic aids in physically active populations [39,40,41].

### 2.9. Statistical Analysis

Statistical analyses were performed using IBM SPSS Statistics (version 27.0; IBM Corp., Armonk, NY, USA). Continuous variables are presented as mean ± standard deviation (SD), whereas categorical variables are presented as frequencies and percentages. The normality of continuous variables was assessed using the Kolmogorov–Smirnov and Shapiro–Wilk tests in conjunction with visual inspection of Q–Q plots. Prior to the factorial analysis of covariance (ANCOVA), the underlying assumptions were evaluated. Homogeneity of variances was assessed using Levene’s test, while the linearity between covariates and dependent variables, homogeneity of regression slopes, and normality of residuals were also examined. No substantial violations of the ANCOVA assumptions were identified. For continuous outcomes, factorial analysis of covariance (ANCOVA) was performed to evaluate the effects of exercise modality (mixed training, Pilates, and CrossFit) and sex, with age and training age included as covariates. These covariates were selected because age and training age are recognized determinants of physical fitness, body composition, and exercise performance and were included to control for their potential confounding effects on the association between exercise modality and the study outcomes. Estimated marginal means (EMMs) and 95% confidence intervals (CIs) were calculated. When a significant main effect of exercise modality was identified, Bonferroni-adjusted pairwise comparisons were performed. Effect sizes were expressed as partial eta squared (ηp^2^). Categorical variables were analyzed using Pearson’s chi-square (χ^2^) test of independence. When the overall comparison among exercise modality groups was statistically significant, Bonferroni-adjusted pairwise chi-square tests were subsequently conducted. Effect sizes were reported as Cramer’s V for comparisons involving more than two groups and the Phi coefficient for 2 × 2 comparisons. All statistical tests were two-tailed, and statistical significance was set at *p* < 0.05.

## 3. Results

### 3.1. Participant Characteristics

The study included 148 recreationally active adults, of whom 52 participated in mixed aerobic–resistance training, 46 in Pilates, and 50 in CrossFit. Participant characteristics according to exercise modality are presented in Table 1. No statistically significant differences were observed among the three exercise-modality groups in age, training age, body mass, height, or body mass index (BMI; all *p* > 0.05). The corresponding effect sizes were small or negligible, indicating that the groups were broadly comparable with respect to their baseline anthropometric and training-related characteristics.

### 3.2. Cardiorespiratory Characteristics

To examine the effects of exercise modality and sex on cardiorespiratory fitness, a factorial analysis of covariance (ANCOVA) was performed with exercise modality (mixed training, Pilates, and CrossFit) and sex as fixed factors and age and training age as covariates. Adjusted estimated marginal means (EMMs), 95% confidence intervals (CIs), and ANCOVA results are presented in Table 2.

Significant effects of exercise modality were observed for most cardiorespiratory outcomes (Table 2). Post hoc Bonferroni-adjusted pairwise comparisons showed that the mixed training and CrossFit groups demonstrated higher relative and absolute VO_2_max than the Pilates group, whereas no significant differences were observed between the mixed training and CrossFit groups. For relative and absolute VO_2_ at VAT, the mixed training group showed significantly higher values than the Pilates group, while the CrossFit group did not differ significantly from either the mixed training or Pilates groups. In contrast, VAT expressed as %VO_2_max was higher in the Pilates group than in the other two exercise modalities.

Significant main effects of sex were identified for relative VO_2_max, absolute VO_2_max, absolute VO_2_ at VAT, and VAT expressed as %VO_2_max, whereas no significant sex differences were observed for relative VO_2_ at VAT or VAT expressed as %HRmax.

A significant exercise modality × sex interaction was observed only for absolute VO_2_max. Follow-up analyses showed a similar pattern of pairwise differences in men and women, suggesting that the interaction reflected differences in the magnitude rather than the direction of the exercise-modality effect. No significant interactions were identified for the remaining cardiorespiratory outcomes.

### 3.3. Body Composition Characteristics

Significant effects of exercise modality were observed for all body composition outcomes examined (Table 3). Post hoc Bonferroni-adjusted pairwise comparisons showed that the Pilates group demonstrated higher fat mass (%), fat mass (kg), and impedance than both the mixed training and CrossFit groups. Conversely, participants in the mixed training and CrossFit groups had higher lean body mass (%) and lean body mass (kg) than those in the Pilates group, with no significant differences between the two exercise modalities. CrossFit participants also showed higher phase angle values than the Pilates group, whereas the mixed training group did not differ significantly from either group.

Significant main effects of sex were identified for fat mass (%), lean body mass (%), phase angle, lean body mass (kg), and impedance. Men had higher lean body mass (%), lean body mass (kg), and phase angle, whereas women had higher fat mass (%) and impedance. Fat mass (kg) did not differ significantly between men and women.

No significant exercise modality × sex interactions were observed for any body composition outcome, suggesting that the effects of exercise modality were consistent between men and women.

### 3.4. Muscular Strength and Flexibility

For muscular strength and flexibility variables, ANCOVA revealed a significant effect of exercise modality only for dominant hand grip strength (Table 4). Post hoc Bonferroni-adjusted pairwise comparisons showed that the mixed training group demonstrated greater dominant hand grip strength than the Pilates group, whereas no significant differences were observed between the mixed training and CrossFit groups or between the Pilates and CrossFit groups. No significant effects of exercise modality were observed for the sit-and-reach or back scratch tests.

Significant main effects of sex were identified for all three outcomes. Men demonstrated greater dominant hand grip strength and better back scratch performance, whereas women achieved higher sit-and-reach scores.

No significant exercise modality × sex interactions were observed for any muscular strength or flexibility outcome, indicating that exercise modality exerted comparable effects across both sexes.

### 3.5. Adherence to the Mediterranean Diet

Mediterranean Diet Score differed according to both exercise modality and sex (Table 5). Post hoc Bonferroni-adjusted pairwise comparisons showed that the Pilates group achieved higher Mediterranean Diet Scores than the mixed training group, whereas no significant differences were observed between the Pilates and CrossFit groups or between the mixed training and CrossFit groups.

A significant exercise modality × sex interaction indicated that the association between exercise modality and adherence to the Mediterranean diet differed between men and women. Estimated marginal means showed similar levels of adherence across exercise modalities among women, whereas among men, the CrossFit group had the lowest Mediterranean Diet Scores and the Pilates group the highest. These findings suggest that the association between exercise modality and adherence to the Mediterranean diet was primarily driven by differences among male participants.

### 3.6. Dietary Supplement Use

Overall, 63 of the 148 participants (42.6%) reported using dietary supplements. Supplement use differed significantly according to exercise modality but not according to sex (Table 6). Post hoc Bonferroni-adjusted pairwise comparisons showed that supplement use was more frequent in the mixed training and CrossFit groups than in the Pilates group, whereas no significant difference was observed between the mixed training and CrossFit groups (Table 7). The prevalence of supplement use was similar in men and women.

### 3.7. Types of Dietary Supplements Used

Protein supplements were the most frequently reported, followed by creatine and vitamins (Table 8). Amino acids were also reported, whereas the remaining supplement categories were uncommon. No participant reported using calcium or enzyme supplements.

### 3.8. Individual Dietary Supplement Use According to Exercise Modality and Sex

The use of protein, creatine, and vitamin supplements did not differ significantly among exercise modalities (Table 9). Significant sex differences were observed for protein and creatine supplement use, both of which were more common among men than women. In contrast, vitamin supplement use did not differ significantly between men and women.

### 3.9. Dietary Supplement Use According to Primary Reason for Use

The distribution of supplement use varied according to the participants’ primary reason for use (Table 10). Protein and creatine supplement use was most frequently reported for performance-related reasons, whereas vitamin and amino acid use was relatively more common among participants who reported appearance-related reasons. Iron supplements were more commonly reported for medical reasons. Given the small number of observations in several categories, these findings should be considered descriptive and interpreted with caution.

## 4. Discussion

The present study compared the fitness, dietary, and supplement-use profiles of recreational adults participating in CrossFit, Pilates, and mixed aerobic-resistance training within organized gym environments. The findings indicate that exercise modality is associated with clear differences in cardiorespiratory fitness, body composition, and muscular strength, whereas flexibility did not differ significantly across groups. Because the participants were assessed within pre-existing exercise groups, the findings should be interpreted as cross-sectional associations rather than as evidence that the exercise modalities caused the observed differences.

One of the main findings was the superior cardiorespiratory profile of CrossFit and mixed training compared to Pilates. Participants engaged in CrossFit and mixed training exhibited higher VO_2_max than the Pilates participants, and this pattern was broadly consistent across sexes. These results are consistent with the physiological demands of CrossFit and mixed training, which typically involve greater aerobic and metabolic stress than Pilates [7,9,11,14,18]. The higher adjusted VO_2_max observed in these groups indicate a more favorable cardiorespiratory profile among recreational adults engaged in these modalities, a finding that is also in line with the broader clinical importance attributed to cardiorespiratory fitness as a health marker [19,42]. This broader practical relevance is in line with recent evidence showing that even brief and feasible forms of physical activity can confer meaningful metabolic, cardiovascular, and functional benefits in adults [43].

At the same time, Pilates participants showed higher VAT values when expressed as a percentage of VO_2_max. This pattern is noteworthy because it suggests that although Pilates participants had lower maximal aerobic capacity, they reached ventilatory threshold at a relatively greater proportion of that capacity. One plausible interpretation is that Pilates may be associated with greater tolerance and control at submaximal intensity levels. However, the pattern of Pilates participants showing higher VAT values when expressed as a percentage of VO_2_max should be interpreted cautiously. Pilates participants also demonstrated lower absolute VO_2_max, while absolute VAT values were not consistently higher across comparisons. Therefore, the higher VAT expressed as %VO_2_max may reflect a relative submaximal profile, at least in part, rather than superior overall aerobic fitness. Given that Pilates participants also showed lower absolute VO_2_max and lower functional capacity, the higher VAT percentage may partly reflect the lower denominator rather than a broader cardiorespiratory advantage. In this context, VAT as %VO_2_max appears to describe a relative submaximal profile, whereas CrossFit and mixed training remained associated with higher maximal cardiorespiratory performance. Importantly, absolute VAT, VO_2_max, and HR-based threshold measures do not support a broad superiority of Pilates in cardiorespiratory function. This interpretation is consistent with the general training philosophy of Pilates and with reports emphasizing its contribution to movement control, functional stability, respiratory regulation, and selected aspects of physical fitness [8,16,17].

Body composition outcomes also showed that participants engaged in CrossFit and mixed aerobic-resistance training exhibited lower fat mass and higher lean body mass than the Pilates participants. The Pilates participants exhibited higher fat mass and impedance than the participants engaged in CrossFit and mixed training. These findings may reflect the lower overall energy expenditure and lower intensity profile generally associated with Pilates compared with modalities incorporating aerobic loading and resistance training. The observation that sex did not significantly influence adjusted fat mass in kilograms suggests that body-composition differences were more strongly associated with exercise modality within this age range. This pattern is compatible with prior reports showing favorable body composition changes in high-intensity functional training and concurrent training settings [14].

Regarding muscular strength, participants engaged in mixed aerobic-resistance training exhibited higher adjusted dominant handgrip strength than the Pilates participants. CrossFit showed intermediate strength values, while Pilates demonstrated the lowest grip strength values in men. In women, however, no significant strength differences were observed across modalities, which may be related to smaller between-group variation or lower modality-specific divergence in upper-body loading. In general, the present findings suggest that resistance-based or mixed exercise models were associated with more favorable strength-related outcomes than lower-intensity movement-focused modalities [1,9,18]. The handgrip findings are consistent with the literature, which suggests that handgrip strength is a valid marker of overall muscular function and functional status, but not as a highly specific indicator of the distinct physiological adaptations elicited by each training modality [44,45]. More specifically, mixed aerobic-resistance exercise has been associated with more favorable handgrip strength values than more unidimensional exercise patterns, which is consistent with the higher handgrip values observed in the mixed training group in the present sample. At the same time, although Pilates is not primarily designed to enhance maximal isometric gripping force, it appears capable of maintaining or improving selected aspects of muscular function and physical performance [16,17], which may explain why the women in the Pilates group were not markedly lower than those in the other groups. In addition, the literature on high-intensity multimodal training modalities such as CrossFit is characterized by substantial heterogeneity in terms of exercise prescription, loading parameters, and outcome assessment [46], and therefore superiority should not be expected across every isolated strength variable. Accordingly, the absence of significant between-group differences in female handgrip strength should not be considered paradoxical, but rather indicative of the fact that this variable may be influenced more strongly by broader biological and functional determinants than by modality-specific training differences alone.

Contrary to common expectations, flexibility did not differ significantly across exercise modalities, as no significant main effect of exercise modality was observed for either the sit-and-reach or the back-scratch test. No significant exercise modality × sex interaction was observed for either flexibility measure, indicating that the absence of modality-related differences was consistent across men and women. Although Pilates is often assumed to confer superior flexibility benefits, this pattern was not statistically confirmed in the present sample. It is possible that recreational participation in all three modalities was sufficient to maintain similar levels of flexibility, or that the selected field tests were not sensitive enough to detect modality-specific mobility adaptations. At the same time, the majority of the Pilates literature supports beneficial effects on flexibility, functional movement, and selected cardiorespiratory indices, but mainly in targeted interventions and specific populations [8,15,16,17,33,47].

The dietary findings provide complementary descriptive context. After adjustment for age and training age, Mediterranean diet adherence differed modestly across exercise modalities, with Pilates participants showing a higher Mediterranean Diet Score than mixed-training participants, while CrossFit participants exhibited intermediate values. A significant exercise modality × sex interaction was also observed. Inspection of the estimated marginal means suggested that this pattern was mainly driven by differences among men, whereas Mediterranean Diet Scores were relatively similar across exercise modalities among women. Mediterranean dietary patterns have been associated with potentially favorable health and performance-related characteristics in physically active populations [48]. However, given the small adjusted main effect of exercise modality and the absence of a consistent pattern across both sexes, the present findings should be interpreted cautiously and should not be considered evidence that participation in a particular exercise modality determines dietary quality. Supplement use has also been reported to be common among athletes and gym-based populations [49].

The results concerning dietary supplement use provide additional descriptive context. A total of 42.6% of the participants used nutritional supplements. Significant differences in overall dietary supplement use were observed across exercise modalities, with a higher prevalence among the mixed-training and CrossFit participants than among the Pilates participants. For descriptive comparison, dietary supplement use in gym-based populations has previously been reported at 36.3% in the Middle East and 37.8% in Saudi Arabia, with protein supplements, omega-3 fatty acids, and multivitamins among the most reported choices [50,51]. In the present sample, protein supplements and creatine were among the most frequently reported products; however, the use of protein, creatine, and vitamin supplements did not differ significantly across exercise modalities. This pattern is in line with the broader sports nutrition literature, where protein, creatine, caffeine, vitamins, and other ergogenic aids are commonly used in physically active and performance-oriented populations [39,40,41,48]. In this sample, overall dietary supplement use differed across exercise modalities, whereas no statistically significant between-modality differences were identified for the individual supplement categories examined. Given the descriptive and non-validated nature of the supplement questionnaire, these findings should be interpreted as sample-specific patterns rather than as evidence of broader behavioral differences between exercise modalities.

The findings about the dietary profile and use of nutritional supplements are important because they indicate that exercise modality may be associated not only with distinct physiological profiles, but also with differences in Mediterranean diet adherence and in the overall prevalence of dietary supplement use. Therefore, the body-composition and performance-related findings should be interpreted with awareness that part of the observed variation may also reflect differences in Mediterranean diet adherence, overall supplement use, and other unmeasured behavioral factors rather than the training stimulus alone [40,48].

The present findings should also not be interpreted exclusively as modality-specific training effects. Differences between groups may have been influenced by non-training factors, including Mediterranean diet adherence, overall dietary supplement use, motivation, exercise adherence, total daily activity, and baseline fitness characteristics that were not included as covariates in the adjusted models. In real-world gym settings, these behavioral and lifestyle-related dimensions are often intertwined with exercise participation and may contribute to the physiological and body-composition profiles observed. For this reason, the present results are better understood as modality-associated profiles within a broader behavioral context rather than as pure effects attributable only to the training stimulus.

Taken together, these results support the view that different gym-based exercise modalities are associated with distinct fitness, dietary, and supplement-use profiles. In addition, the present study contributes novelty to the literature by offering, to the best of our knowledge, one of the few direct simultaneous comparisons of CrossFit, Pilates, and mixed aerobic–resistance training across a broad set of cardiorespiratory, body composition, strength, and flexibility indicators within the same recreational adult sample. CrossFit and mixed-training participants exhibited higher cardiorespiratory fitness, lower fat mass, and higher lean body mass than the Pilates participants. Mixed training was also associated with higher adjusted dominant handgrip strength than Pilates, whereas CrossFit showed intermediate values and flexibility did not differ significantly across modalities. Pilates participants showed a higher VAT expressed relative to VO_2_max, although this finding was not accompanied by higher absolute cardiorespiratory performance and should be interpreted as a relative submaximal profile. Mediterranean diet adherence showed modest and sex-specific differences, while overall supplement use was less frequent among the Pilates participants than among the CrossFit and mixed-training participants. Therefore, exercise prescription should be individualized according to specific health, fitness, and behavioral goals.

Moreover, the study is characterized by several important methodological strengths. First, the relatively large and balanced sample of healthy adults of both sexes enhances statistical power and allows for the simultaneous examination of the main effects of exercise modality and sex, as well as the exercise modality × sex interaction. Furthermore, the multifactorial approach, incorporating anthropometric, cardiorespiratory, and functional indices, provides a comprehensive characterization of the physiological profile of the participants. Additional value also lies in the comparison of three contemporary and widely practiced exercise modalities under real-world fitness center conditions, thereby enhancing the ecological validity of the findings and their applicability to both training and clinical practice. Importantly, the comparative role of the study should be particularly emphasized, as it addresses a clear gap in the literature by directly comparing three of the most popular contemporary exercise modalities practiced in gym settings—CrossFit, Pilates, and mixed training—within the same methodological framework. This study provides a direct simultaneous examination of these modalities in a recreational adult sample using a multidimensional set of functional indicators. In this respect, its contribution lies in the integrated comparison of these modalities within the same methodological framework and in the characterization of their associated physiological and lifestyle-related profiles.

Despite these strengths, several limitations should be acknowledged. First, the cross-sectional design precludes causal inference and does not allow for the evaluation of long-term training adaptations. Second, participants were not randomly assigned to exercise modality but were assessed according to their pre-selected training model, introducing the possibility of self-selection bias. Accordingly, some of the observed between-group differences may reflect pre-existing characteristics, preferences, motivations, or capacities that influenced exercise choice rather than the independent effect of the modality itself. In addition, the absence of baseline pre-training values limits the ability to determine whether the observed differences represent training-related adaptations or pre-existing physiological and behavioral characteristics. Another possible source of measurement variability is that the participants did not complete a separate familiarization or pilot session before the treadmill test. Moreover, variation in total training load, previous training background, prior fitness level, and overall exercise exposure may also have contributed to the observed differences between groups. Exact individual weekly training volume, training load, and progression over time were not systematically recorded. In addition, an identical standardized training protocol was not implemented across all participating facilities. These factors may have contributed to variability in training exposure within each exercise-modality group. Although a wide range of physiological parameters was assessed, long-term adherence, precise training load, detailed dietary intake, overall physical activity, and previous sport experience were not included as covariates in the adjusted statistical models. Body-composition findings should also be interpreted with caution, given the field-based limitations of bioelectrical impedance analysis and the absence of detailed dietary energy-intake data. Mediterranean diet adherence and overall and category-specific dietary supplement use were assessed by self-report and are therefore subject to recall bias and reporting inaccuracies. Moreover, although Mediterranean diet adherence was assessed using the validated MedDietScore, the supplement-related section was developed specifically for the present study as a descriptive data-collection tool and had not undergone formal validation. Consequently, the supplement-use findings represent descriptive, sample-specific patterns rather than validated measures of supplement-use behavior. Taken together, these considerations indicate that the present findings reflect modality-associated profiles rather than confirmed effects attributable solely to the training stimulus. The findings of the present study should be interpreted within the context of these methodological considerations, while future longitudinal and randomized interventional studies may further expand the understanding of adaptations associated with contemporary forms of exercise.

## 5. Conclusions

In this cross-sectional sample, participants engaged in CrossFit and mixed aerobic–resistance training exhibited higher adjusted cardiorespiratory fitness, lower fat mass, and higher lean body mass than the Pilates participants. Pilates participants exhibited a higher VAT expressed relative to VO_2_max; however, this finding reflects a relative submaximal profile and should not be interpreted as superior overall aerobic fitness. Mixed-training participants also exhibited higher adjusted dominant handgrip strength than the Pilates participants, whereas CrossFit participants showed intermediate values, and flexibility did not differ significantly across exercise modalities. Mediterranean diet adherence showed modest and sex-specific differences after adjustment for age and training age. Dietary supplement use differed across exercise modalities at the overall level, whereas no statistically significant between-modality differences were identified for the individual supplement categories examined; therefore, these findings should be interpreted as descriptive and sample-specific. Overall, recreational adults practicing CrossFit or mixed aerobic–resistance training displayed higher cardiorespiratory fitness and more favorable body composition than those practicing Pilates, while Pilates showed a higher VAT relative to VO_2_max; however, these differences should be interpreted as modality-associated profiles rather than proven training effects.

## Figures and Tables

**Table 1:** Participant characteristics according to exercise modality.

Variable	Mixed Training (*n* = 52)	Pilates (*n* = 46)	CrossFit (*n* = 50)	*p*	η^2^
	Mean ± SD	Mean ± SD	Mean ± SD		
Age (years)	33.67 ± 6.01	33.80 ± 7.26	32.02 ± 5.80	0.301	0.02
Training age (years)	3.56 ± 2.16	2.98 ± 1.86	3.52 ± 2.17	0.316	0.02
Body mass (kg)	74.58 ± 15.54	73.07 ± 14.08	72.67 ± 13.00	0.777	0.00
Height (cm)	174.54 ± 8.84	173.89 ± 7.67	172.32 ± 10.40	0.451	0.01
BMI (kg/m^2^)	24.29 ± 3.61	24.08 ± 3.86	24.30 ± 2.53	0.937	0.00

**Notes:** Data are presented as mean ± standard deviation (SD). BMI, body mass index. Group differences were examined using one-way analysis of variance (ANOVA). η^2^, eta-squared effect size.

**Table 2:** Estimated marginal means (EMMs) for the cardiorespiratory fitness variables and the results of factorial ANCOVA according to exercise modality and sex.

Variable	Mixed Training EMM (95% CI)	Pilates EMM (95% CI)	CrossFit EMM (95% CI)	Exercise Modality F; *p*; ηp^2^	Sex F; *p*; ηp^2^	Modality × Sex F; *p*; ηp^2^
Relative VO_2_max (mL·kg^−1^·min^−1^)	44.03 ^a^ (42.43–45.62)	33.90 ^b^ (32.20–35.60)	44.97 ^a^ (43.36–46.58)	38.24; <0.001; 0.35	18.03; <0.001; 0.11	2.47; 0.089; 0.03
Absolute VO_2_max (mL·min^−1^)	3282.60 ^a^ (3128.17–3437.04)	2496.66 ^b^ (2333.05–2660.27)	3269.39 ^a^ (3113.90–3424.88)	35.73; <0.001; 0.34	189.04; <0.001; 0.58	4.25; 0.016; 0.06
Relative VO_2_ at VAT (mL·kg^−1^·min^−1^)	23.09 ^a^ (22.11–24.06)	20.50 ^b^ (19.47–21.54)	22.12 ^ab^ (21.13–23.10)	7.55; 0.001; 0.10	0.20; 0.653; 0.00	2.50; 0.086; 0.03
Absolute VO_2_ at VAT (mL·min^−1^)	1721.28 ^a^ (1645.94–1796.63)	1504.37 ^b^ (1424.52–1584.21)	1592.59 ^ab^ (1516.77–1668.42)	7.59; 0.001; 0.10	86.04; <0.001; 0.38	2.89; 0.059; 0.04
VAT (%VO_2_max)	52.97 ^b^ (51.52–54.41)	61.60 ^a^ (60.07–63.13)	49.48 ^b^ (48.03–50.93)	18.23; <0.001; 0.21	15.55; <0.001; 0.10	0.25; 0.780; 0.00
VAT (%HRmax)	67.63 ^a^ (65.87–69.40)	69.63 ^a^ (67.76–71.50)	66.61 ^a^ (64.83–68.39)	0.58; 0.562; 0.01	0.28; 0.598; 0.00	0.55; 0.581; 0.01

**Notes:** Data are presented as estimated marginal means (EMMs) with 95% confidence intervals (CIs), adjusted for age and training age. Factorial ANCOVA included exercise modality (mixed training, Pilates, and CrossFit) and sex as fixed factors. Different superscript letters within the same row indicate significant Bonferroni-adjusted pairwise differences (*p* < 0.05); values sharing at least one superscript letter do not differ significantly. ANCOVA, analysis of covariance; EMM, estimated marginal mean; HRmax, maximal heart rate; VAT, ventilatory anaerobic threshold; VO_2_, oxygen uptake; VO_2_max, maximal oxygen uptake; ηp^2^, partial eta squared.

**Table 3:** Estimated marginal means (EMMs) for the body composition variables and results of factorial ANCOVA according to exercise modality and sex.

Variable	Mixed Training EMM (95% CI)	Pilates EMM (95% CI)	CrossFit EMM (95% CI)	Exercise Modality F; *p*; ηp^2^	Sex F; *p*; ηp^2^	Modality × Sex F; *p*; ηp^2^
Fat mass (%)	18.65 ^b^ (16.94–20.36)	24.89 ^a^ (23.05–26.72)	18.67 ^b^ (16.90–20.44)	15.38; <0.001; 0.18	45.49; <0.001; 0.25	0.53; 0.587; 0.01
Lean body mass (%)	81.63 ^a^ (79.83–83.43)	75.18 ^b^ (73.26–77.10)	81.35 ^a^ (79.50–83.19)	14.46; <0.001; 0.17	46.09; <0.001; 0.25	0.89; 0.413; 0.01
Phase angle (°)	7.23 ^ab^ (6.71–7.74)	6.35 ^b^ (5.81–6.89)	7.46 ^a^ (6.94–7.98)	4.66; 0.011; 0.06	4.07; 0.046; 0.03	0.54; 0.583; 0.01
Fat mass (kg)	14.00 ^b^ (12.27–15.73)	18.65 ^a^ (16.81–20.49)	13.22 ^b^ (11.44–14.99)	10.15; <0.001; 0.13	0.67; 0.413; 0.01	0.71; 0.492; 0.01
Lean body mass (kg)	60.90 ^a^ (59.11–62.68)	55.60 ^b^ (53.68–57.52)	59.03 ^a^ (57.18–60.88)	8.07; <0.001; 0.10	389.53; <0.001; 0.74	1.41; 0.249; 0.02
Impedance (Ω)	498.61 ^b^ (478.69–518.54)	564.83 ^a^ (544.08–585.58)	483.36 ^b^ (463.39–503.33)	17.22; <0.001; 0.20	70.97; <0.001; 0.34	0.74; 0.480; 0.01

**Notes:** Data are presented as estimated marginal means (EMMs) with 95% confidence intervals (CIs), adjusted for age and training age. Factorial ANCOVA included exercise modality (mixed training, Pilates, and CrossFit) and sex as fixed factors. Different superscript letters within the same row indicate significant Bonferroni-adjusted pairwise differences (*p* < 0.05); values sharing at least one superscript letter do not differ significantly.

**Table 4:** Estimated marginal means (EMMs) for the muscular strength and flexibility variables and results of factorial ANCOVA according to exercise modality and sex.

Variable	Mixed Training EMM (95% CI)	Pilates EMM (95% CI)	CrossFit EMM (95% CI)	Exercise Modality F; *p*; ηp^2^	Sex F; *p*; ηp^2^	Modality × Sex F; *p*; ηp^2^
Dominant hand grip strength (kg)	44.32 ^a^ (42.36–46.27)	40.26 ^b^ (38.16–42.36)	41.16 ^ab^ (39.16–43.17)	4.43; 0.014; 0.06	249.93; <0.001; 0.64	2.76; 0.067; 0.04
Sit-and-reach (cm)	7.60 ^a^ (5.20–9.99)	4.20 ^a^ (1.63–6.78)	7.15 ^a^ (4.69–9.60)	2.07; 0.130; 0.03	33.48; <0.001; 0.19	0.06; 0.939; 0.00
Back scratch (cm)	5.47 ^a^ (3.26–7.69)	5.18 ^a^ (2.73–7.63)	2.47 ^a^ (0.20–4.74)	2.04; 0.133; 0.03	17.76; <0.001; 0.11	1.09; 0.339; 0.02

**Notes:** Data are presented as estimated marginal means (EMMs) with 95% confidence intervals (CIs), adjusted for age and training age. Factorial ANCOVA included exercise modality (mixed training, Pilates, and CrossFit) and sex as fixed factors. Different superscript letters within the same row indicate significant Bonferroni-adjusted pairwise differences (*p* < 0.05); values sharing at least one superscript letter do not differ significantly.

**Table 5:** Estimated marginal means (EMMs) for Mediterranean Diet Score and results of factorial ANCOVA according to exercise modality and sex.

Variable	Mixed Training EMM (95% CI)	Pilates EMM (95% CI)	CrossFit EMM (95% CI)	Exercise Modality F; *p*; ηp^2^	Sex F; *p*; ηp^2^	Modality × Sex F; *p*; ηp^2^
Mediterranean Diet Score	30.23 ^b^ (29.11–31.35)	32.29 ^a^ (31.09–33.49)	31.29 ^ab^ (30.14–32.44)	3.08; 0.049; 0.04	14.34; <0.001; 0.09	7.32; 0.001; 0.10

**Notes:** Data are presented as estimated marginal means (EMMs) with 95% confidence intervals (CIs), adjusted for age and training age. Factorial ANCOVA included exercise modality (mixed training, Pilates, and CrossFit) and sex as fixed factors. Different superscript letters indicate significant Bonferroni-adjusted pairwise differences (*p* < 0.05); values sharing at least one superscript letter do not differ significantly.

**Table 6:** Overall dietary supplement use according to exercise modality and sex.

Variable	Category	No, n (%)	Yes, n (%)	χ^2^ (df)	*p*	Effect Size
Exercise modality	Mixed training (*n* = 52)	21 (40.4)	31 (59.6)	27.76 (2)	<0.001	Cramer’s V = 0.43
	Pilates (*n* = 46)	41 (89.1)	5 (10.9)			
	CrossFit (*n* = 50)	23 (46.0)	27 (54.0)			
Sex	Men (*n* = 72)	41 (56.9)	31 (43.1)	0.01 (1)	0.907	Phi = 0.01
	Women (*n* = 76)	44 (57.9)	32 (42.1)			

**Notes:** Values are presented as number of participants (*n*) and percentages (% within each group). Pearson’s chi-square test was used to compare the overall dietary supplement use. Effect sizes are reported as Cramer’s V (exercise modality) and Phi coefficient (sex).

**Table 7:** Pairwise comparisons of overall dietary supplement use between exercise modalities.

Comparison	χ^2^ (df)	*p*	Phi
Mixed training vs. Pilates	24.96 (1)	<0.001	0.51
Mixed training vs. CrossFit	0.33 (1)	0.567	0.06
Pilates vs. CrossFit	20.06 (1)	<0.001	0.46

**Notes:** Pairwise comparisons were performed using Pearson’s chi-square tests with Bonferroni correction for multiple comparisons. Phi coefficients are reported as absolute values.

**Table 8:** Prevalence of individual dietary supplement use.

Dietary Supplement	n (%)
Proteins	43 (29.1)
Creatine	23 (15.5)
Vitamins	22 (14.9)
Amino acids	9 (6.1)
Iron	7 (4.7)
Caffeine	6 (4.1)
Isotonic drinks	5 (3.4)
Energy drinks	5 (3.4)
Carbohydrates	3 (2.0)
Other minerals	1 (0.7)
Carnitine	1 (0.7)
Glutamine	1 (0.7)

**Notes:** Values are presented as the number of participants (*n*) and percentage (%) of the total sample (*N* = 148) reporting the use of each dietary supplement. Participants could report the use of more than one dietary supplement; therefore, percentages do not sum 100%.

**Table 9:** Individual dietary supplement use according to exercise modality and sex.

Dietary Supplement	Exercise Modality			χ^2^ (df)	*p*	Cramer’s V	Sex		χ^2^ (df)	*p*	Phi
	Mixed	Pilates	CrossFit				Men	Women			
Protein	12 (23.1)	12 (26.1)	19 (38.0)	3.04 (2)	0.219	0.14	30 (41.7)	13 (17.1)	10.82 (1)	0.001	0.27
Creatine	8 (15.4)	6 (13.3)	9 (18.0)	0.40 (2)	0.821	0.05	16 (22.5)	7 (9.2)	4.94 (1)	0.026	0.18
Vitamins	5 (9.6)	11 (23.9)	6 (12.0)	4.43 (2)	0.109	0.17	13 (18.1)	9 (11.8)	1.13 (1)	0.288	0.09

**Notes:** Values are presented as the number of participants (*n*) and percentages (% within each exercise modality or sex). Pearson’s chi-square test was used for all comparisons. Effect sizes are reported as Cramer’s V for comparisons among exercise modalities and Phi coefficient for comparisons between sexes.

**Table 10:** Distribution of dietary supplement use according to primary reason for use.

Dietary Supplement	Performance n (%)	Appearance n (%)	Medical n (%)
Proteins	14 (38.9)	5 (31.3)	2 (18.2)
Creatine	8 (22.2)	3 (18.8)	1 (9.1)
Vitamins	7 (19.4)	5 (31.3)	0 (0.0)
Amino acids	5 (13.9)	3 (18.8)	0 (0.0)
Iron	2 (5.6)	1 (6.3)	2 (18.2)
Caffeine	2 (5.6)	1 (6.3)	0 (0.0)
Carbohydrates	2 (5.6)	0 (0.0)	0 (0.0)

**Notes:** Values are presented as the number of participants (*n*) and percentages (% within each primary reason for supplement use). Percentages are calculated using the total number of participants reporting each primary reason for supplement use. Owing to the small number of observations in several categories, the table is presented for descriptive purposes only.

## Data Availability

The data presented in this study are available on request from the corresponding author. The data are not publicly available due to privacy and ethical restrictions.
